# Feasibility of atezolizumab and bevacizumab combination regimens in patients with hepatocellular carcinoma and lung cancer taking direct oral anticoagulants

**DOI:** 10.1002/cam4.7430

**Published:** 2024-06-25

**Authors:** Tasuku Nakabori, Kei Kunimasa, Masaki Kawabata, Sena Higashi, Kaori Mukai, Takahisa Kawamura, Takako Inoue, Motohiro Tamiya, Kazumi Nishino, Kazuyoshi Ohkawa

**Affiliations:** ^1^ Department of Hepatobiliary and Pancreatic Oncology Osaka International Cancer Institute Osaka Japan; ^2^ Department of Thoracic Oncology Osaka International Cancer Institute Osaka Japan

**Keywords:** atezolizumab and bevacizumab, bleeding event, combination immunotherapy, direct oral anticoagulant, hepatocellular carcinoma, lung cancer

## Abstract

**Aim:**

Atezolizumab and bevacizumab (Atezo/Bev) combination immunotherapy regimens and direct oral anticoagulants (DOACs) are both associated with bleeding. Therefore, combining Atezo/Bev regimens with DOACs may exacerbate the bleeding risk. This study investigated the feasibility of the Atezo/Bev regimen in patients taking DOACs.

**Methods:**

This retrospective study included 141 patients with unresectable hepatocellular carcinoma (HCC) or advanced lung cancer (LC) treated with Atezo/Bev regimens. Patients who used antithrombotic agents other than DOACs were excluded. Bleeding events during the Atezo/Bev regimen were analyzed.

**Results:**

The incidence rates of bleeding of any grade in the DOAC (*n* = 11) and no antithrombotic agent (NAA) (*n* = 130) groups were 9.1% and 10.8%, respectively, with no significant differences. Moreover, no significant difference was found in the frequency of bleeding of grade ≥3 between the DOAC and NAA groups. No patients in the DOAC group discontinued the Atezo/Bev regimen because of severe bleeding. Although serum albumin levels, with a hazard ratio (HR) of 0.298 (95% confidence interval [CI]: 0.105–0.847), independently contributed to bleeding events (*p* = 0.023), DOAC administration did not (HR: 1.357; 95% CI: 0.157–10.54; *p* = 0.770). Among only patients with HCC (*n* = 59), none of the five patients taking DOACs experienced bleeding events. A high albumin–bilirubin score (HR: 9.083, 95% CI: 1.118–73.76) was associated with bleeding events (*p* = 0.039).

**Conclusions:**

DOACs did not have a considerable effect on bleeding events in the Atezo/Bev regimens for HCC or LC. Under careful surveillance for bleeding, Atezo/Bev regimens may be feasible in patients receiving DOACs.

## Graphical abstract

Potential exacerbation of the bleeding risk in atezolizumab and bevacizumab (Atezo/Bev)‐based regimens in patients taking direct oral anticoagulants (DOACs) is a concern. This study analyzed the feasibility of Atezo/Bev regimens in patients with hepatocellular carcinoma (HCC) and lung cancer (LC) taking DOACs. Since DOACs did not have a considerable effect on bleeding events in the Atezo/Bev regimens for HCC or LC, Atezo/Bev regimens may be feasible for patients receiving DOACs under careful surveillance for bleeding.

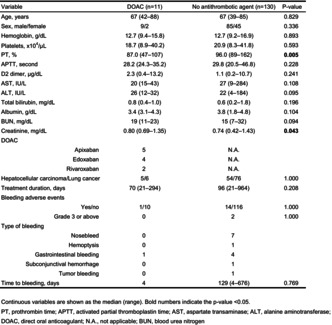

## 1 INTRODUCTION

Direct oral anticoagulants (DOACs) selectively inhibit thrombin (dabigatran) or activated factor X (apixaban, rivaroxaban, and edoxaban). DOACs are widely used to prevent ischemic stroke or systemic embolism in patients with non‐valvular atrial fibrillation or for treating deep vein thrombosis instead of conventional anticoagulants, such as vitamin K antagonists or low‐molecular‐weight heparin (LMWH). This is because of several specific characteristics, including immediate effects, convenience with no coagulation test monitoring required, and weak interaction with drugs and food.^1^, ^2^ Patients with cancer are also increasingly taking DOACs^3^, ^4^; however, bleeding events are a concern because of the greater incidence of major bleeding in patients with cancer with the use of DOACs compared with the use of LMWH^5^ and limited available data on the bleeding risks of DOACs in combination with anticancer drugs.

Bevacizumab is a recombinant humanized monoclonal antibody targeting vascular endothelial growth factor (VEGF). It is thought to augment the antitumor effects of immune checkpoint inhibitors by restoring VEGF‐mediated immunosuppression, such as the inhibition of dendritic cell maturation or proliferation of regulatory T cells and myeloid‐derived suppressor cells, or by promoting the intratumoral infiltration of activated CD8‐positive T cells by normalization of tumor vessels.^6^, ^7^ Recently, atezolizumab and bevacizumab (Atezo/Bev) combination immunotherapy with or without cytotoxic agents has been found to favorably improve the prognosis of unresectable hepatocellular carcinoma (HCC) and advanced lung cancer (LC) and has been approved for treatment.^8^, ^9^ However, bevacizumab poses a risk of bleeding because the disturbance in the VEGF pathway could also suppress the proliferation of vascular endothelial cells, angiogenesis, and interactions between platelets.^10^, ^11^

Bleeding is a concern associated with the concomitant use of the Atezo/Bev combination immunotherapy and DOAC, both of which show a high bleeding risk. In the first‐line treatment of unresectable HCC, patients with a high tendency for bleeding are regarded as bevacizumab‐unfit, and an alternative treatment regimen (durvalumab and tremelimumab) to Atezo/Bev treatment has been proposed.^12^, ^13^ For predisposition to bleeding by antithrombotic agents, aspirin use was not regarded as a high‐risk factor for severe bleeding^14^; however, no detailed studies on DOACs have been conducted to date.

To address this deficiency in the literature, this study analyzed the effect of DOAC on bleeding events in patients with unresectable HCC or advanced LC during Atezo/Bev‐based regimens.

## 2 METHODS

### 2.1 Study population

We retrospectively collected the clinical data of patients with unresectable HCC or advanced LC who were treated with Atezo/Bev‐based regimens at the Osaka International Cancer Institute, Osaka, Japan, between January 2019 and July 2023. The administered regimens were 1200 mg atezolizumab plus 15 mg/kg bevacizumab every 3 weeks for unresectable HCC or four to six courses of 1200 mg atezolizumab plus 15 mg/kg bevacizumab in combination with a platinum doublet including carboplatin and paclitaxel or carboplatin and pemetrexed every 3 weeks, followed by maintenance therapy of 1200 mg atezolizumab plus 15 mg/kg bevacizumab every 3 weeks for advanced LC. Patients taking antithrombotic agents other than DOACs (e.g., aspirin, warfarin, clopidogrel, or LMWH) were excluded. The initiation date of Atezo/Bev‐based regimens was defined at the start of the follow‐up. The end of follow‐up was defined as the end date of the Atezo/Bev‐based regimens. The data cut‐off date was September 30, 2023. This study was conducted in accordance with the Declaration of Helsinki and approved by the Institutional Review Board for Clinical Research at the Osaka International Cancer Institute (approval number: 23200), which waived the requirement for informed consent. The opt‐out method for informed consent was provided to the patients via our hospital website.

### 2.2 Assessment of bleeding events

The diagnosis of bleeding events was made by two physicians: one was the attending doctor of the patient and the other was an author of the study (T.N. or S.H.). The severity of the bleeding events was retrospectively graded according to the National Cancer Institute Common Terminology Criteria for Adverse Events (version 5.0).

### 2.3 Statistical analysis

Continuous variables are expressed as medians (ranges) and were compared using the Mann–Whitney *U*‐test or Wilcoxon signed‐rank test, as appropriate. Categorical variables are expressed as numbers and were compared using Pearson's chi‐squared test or Fisher's exact test, as appropriate. Gray's test was used to compare the cumulative incidence of bleeding. Hazard ratios and 95% confidence intervals (CIs) were determined using multivariate Cox proportional hazards modeling. Differences were considered statistically significant at *p* < 0.05. All statistical analyses were performed using EZR (Saitama Medical Center, Jichi Medical University, Saitama, Japan),^15^ a graphical interface for the R commander software package for Windows (version 1.61; R Foundation for Statistical Computing, Vienna, Austria).

## 3 RESULTS

### 3.1 Bleeding events in patients taking DOACs and those not taking any antithrombotic agents

This study enrolled 141 patients. The baseline characteristics of all patients at the time of initiation of Atezo/Bev‐based regimens are shown in Table 1. All 11 patients, nine receiving treatment for deep vein thrombosis and two for non‐valvular atrial fibrillation, had begun taking DOACs prior to the initiation of Atezo/Bev‐based regimens. None of the patients taking DOACs experienced worsened thrombosis or embolism. None of the patients discontinued DOACs before the end of the Atezo/Bev regimens. Baseline characteristics of the DOAC and no antithrombotic agent (NAA) groups were compared. No significant differences were found in age, sex ratio, platelet count, activated partial thromboplastin time (APTT), D2 dimer level, liver enzymes, serum albumin level, or ratio of HCC to LC between the DOAC and NAA groups. Prothrombin time (PT) was shorter (*p* = 0.005) and serum creatinine level was higher (*p* = 0.043) in the DOAC group than that in the NAA group.

**TABLE 1:** Comparison of characteristics, treatment regimens and bleeding events of patients with DOAC or without any antithrombotic agents.

Variable	DOAC (*n* = 11)	No antithrombotic agent (*n* = 130)	*p*‐value
Age, years	67 (42–88)	67 (39–85)	0.829
Sex, male/female	9/2	85/45	0.336
Hemoglobin, g/dL	12.7 (9.4–15.8)	12.7 (9.2–16.9)	0.893
Platelets, ×10^4^/μL	18.7 (8.9–40.2)	20.9 (8.3–41.8)	0.593
PT, %	87.0 (47–107)	96.0 (89–162)	**0.005**
APTT, second	28.2 (24.3–35.2)	29.8 (20.5–46.8)	0.228
D2 dimer, μg/dL	2.3 (0.4–13.2)	1.1 (0.2–10.7)	0.241
AST, IU/L	20 (15–43)	27 (9–284)	0.108
ALT, IU/L	26 (12–32)	22 (4–184)	0.095
Total bilirubin, mg/dL	0.8 (0.4–1.0)	0.6 (0.2–1.8)	0.196
Albumin, g/dL	3.4 (3.1–4.3)	3.8 (1.8–4.8)	0.104
BUN, mg/dL	19 (11–23)	15 (7–32)	0.094
Creatinine, mg/dL	0.80 (0.69–1.35)	0.74 (0.42–1.43)	**0.043**
DOAC			
Apixaban	5	N.A.	
Edoxaban	4	N.A.	
Rivaroxaban	2	N.A.	
Hepatocellular carcinoma/lung cancer	5/6	54/76	1.000
Treatment duration, days	70 (21–294)	96 (21–964)	0.208
Bleeding adverse events			
Yes/no	1/10	14/116	1.000
Grade 3 or above	0	2	1.000
Type of bleeding			
Nosebleed	0	7	
Hemoptysis	0	1	
Gastrointestinal bleeding	1	4	
Subconjunctival hemorrhage	0	1	
Tumor bleeding	0	1	
Time to bleeding, days	4	129 (4–676)	0.769

*Note*: Continuous variables are shown as the median (range). Bold numbers indicate the *p*‐value <0.05.

Abbreviations: ALT, alanine aminotransferase; APTT, activated partial thromboplastin time; AST, aspartate transaminase; BUN, blood urea nitrogen; DOAC, direct oral anticoagulant; N.A., not applicable; PT, prothrombin time.

Treatment‐related factors and bleeding events were compared. No difference was found in the treatment duration between the DOAC and NAA groups. One (9.1%) and 14 (10.8%) patients in the DOAC and NAA groups, respectively, experienced bleeding events. Among them, bleeding events of grade ≥3 occurred in no patients and two patients in the DOAC and NAA groups, respectively. No significant differences were found in the frequency of bleeding events of any grade or grade ≥3 between the DOAC and NAA groups. The cumulative incidences of bleeding events were also compared (Figure S1). No significant difference was found between the two groups (*p* = 0.772).

### 3.2 Analysis of factors contributing to bleeding events

The factors contributing to bleeding events were analyzed. The two factors with the lowest *p*‐values, PT (*p* = 0.117) and serum albumin level (*p* = 0.005), were included in the multivariate logistic regression model (Table 2). Serum albumin level remained independently associated with bleeding events (*p* = 0.023). The hazard ratio for DOAC administration was 1.357 (95% CI: 0.175–10.54), with no clinical significance (*p* = 0.770).

**TABLE 2:** Factors contributing to bleeding.

	Univariate analysis			Multivariate analysis		
	HR	95% CI	*p*‐value	HR	95% CI	*p*‐value
Age, <65 versus ≥65 years	1.240	0.422–3.644	0.696			
Sex, male versus female	1.212	0.329–4.466	0.773			
Platelets	1.016	0.966–1.069	0.533			
PT	1.028	0.993–1.065	0.117	0.993	0.954–1.033	0.714
APTT	1.013	0.907–1.132	0.814			
D2 dimer	0.952	0.911–1.091	0.814			
AST, <40 versus ≥40 IU/L	1.534	0.539–4.370	0.423			
ALT, <40 versus ≥40 IU/L	1.915	0.423–8.696	0.398			
Albumin	0.270	0.108–0.675	**0.005**	0.298	0.105–0.847	**0.023**
Creatinine, <1.0 versus ≥1.0 mg/dL	1.267	0.354–4.528	0.716			
DOAC administration, yes versus no	1.357	0.175–10.54	0.770			
Hepatocellular carcinoma versus lung cancer	0.627	0.205–1.918	0.413			

*Note*: Bold numbers indicate the *p*‐value <0.05.

Abbreviations: ALT, alanine aminotransferase; APTT, activated partial thromboplastin time; AST, aspartate transaminase; CI, confidence interval; DOAC, direct oral anticoagulant; HR, hazard ratio; PT, prothrombin time.

### 3.3 Comparison of bleeding events in only patients with unresectable HCC taking DOACs and those not taking any antithrombotic agents

Only patients with unresectable HCC (*n* = 59) were included in this analysis. The baseline characteristics at the time of initiation of Atezo/Bev treatment in patients with HCC are shown in Table 3. Five patients were administered DOACs at the initiation of Atezo/Bev treatments. No significant differences were found in age, sex ratio, platelet count, PT, APTT, D2 dimer level, serum albumin level, or serum creatinine level between the DOAC and NAA groups. The aspartate transaminase and alanine aminotransferase levels were lower in the DOAC group than those in the NAA group. No significant differences were found between the two groups in hepatic reserve, tumor‐related factors, including tumor markers and stage, and esophageal varices. No patients in the DOAC group experienced bleeding events. Eight patients (14.8%) in the NAA group experienced bleeding events; two of them experienced bleeding events of grade ≥3. No significant differences were found in the frequency of bleeding events between the two groups. Factors associated with bleeding events were also analyzed (Table 4). A high albumin–bilirubin (ALBI) score, with a hazard ratio of 9.083 (95% CI: 1.118–73.76), contributed to bleeding events (*p* = 0.039).

**TABLE 3:** Comparison of characteristics and treatment of patients with hepatocellular carcinoma with DOAC or without any antithrombotic agents.

Variable	DOAC (*n* = 5)	No antithrombotic agent (*n* = 54)	*p*‐value
Age, years	69 (66–88)	72 (46–85)	0.967
Sex, male/female	5/0	46/8	0.808
Hemoglobin, g/dL	12.2 (11.6–14.1)	12.5 (9.2–16.9)	0.828
Platelets, ×10^4^/μL	18.7 (8.9–12.0)	20.9 (8.3–41.8)	0.056
PT, %	89.0 (47–107)	94.0 (89–162)	0.340
APTT, second	27.4 (25.2–35.2)	29.7 (21.0–40.2)	0.089
D2 dimer, μg/dL	1.8 (0.6–4.8)	1.3 (0.2–7.2)	0.531
AST, IU/L	20 (15–43)	37 (18–284)	**0.028**
ALT, IU/L	14 (12–20)	26 (10–184)	**0.008**
Total bilirubin, mg/dL	0.8 (0.4–1.0)	0.7 (0.3–1.8)	0.845
Albumin, g/dL	3.5 (3.1–4.3)	3.7 (2.7–4.6)	0.651
BUN, mg/dL	22 (16–23)	15 (8.0–29)	**0.017**
Creatinine, mg/dL	1.12 (0.69–1.35)	0.78 (0.42–1.42)	0.093
ALBI grade, 1/2a/2b	2/0/3	20/16/18	0.420
ALBI score	−2.225 (−2.849 to −2.020)	−2.501 (−3.243 to −1.454)	0.688
BCLC stage, A/B/C	1/1/3	1/13/40	0.176
Serum AFP, ng/mL	9 (3–251)	17 (<2– >100,000)	0.703
Serum DCP, mAU/mL	73 (65–11,539)	335 (<30–220,629)	0.693
Esophageal varix, F0/1/2/3	3/1/1/0	35/14/5/0	0.606
Treatment or rupture history of esophageal varix, yes/no	1/4	3/51	0.310
DOAC			
Apixaban	1	N.A.	
Edoxaban	3	N.A.	
Rivaroxaban	1	N.A.	
Atezolizumab+bevacizumab treatment duration, days	202 (70–294)	168 (21–964)	0.978
Bleeding adverse events			
Yes/no	0/5	8/46	0.808
Grade 3 or above	0	2	1.000
Type of bleeding			
Nosebleed	0	4	
Gastrointestinal bleeding	0	2	
Subconjunctival hemorrhage	0	1	
Tumor bleeding	0	1	
Time to bleeding, days	N.A.	201 (4–676)	

*Note*: Continuous variables are shown as the median (range). Bold numbers indicate the *p*‐value <0.05.

Abbreviations: AFP, α‐fetoprotein; ALBI, albumin‐bilirubin; ALT, alanine aminotransferase; APTT, activated partial thromboplastin time; AST, aspartate transaminase; DCP, des‐γ‐carboxy prothrombin; DOAC, direct oral anticoagulant; N.A., not applicable; PT, prothrombin time.

**TABLE 4:** Factors associated with bleeding in patients with hepatocellular carcinoma.

	Univariate analysis		
	HR	95% CI	*p*‐value
Age, <65 versus ≥65 years	0.759	0.179–3.217	0.708
Sex, male versus female	1.008	0.116–8.730	0.994
Platelets	1.032	0.943–1.129	0.500
PT	1.009	0.951–1.071	0.762
APTT	1.070	0.916–1.250	0.397
D2 dimer	1.030	0.595–1.783	0.915
AST, <40 versus ≥40 IU/L	3.705	0.739–18.57	0.111
ALT, <40 versus ≥40 IU/L	2.406	0.298–20.49	0.422
Albumin	0.212	0.034–1.311	0.095
Creatinine, <1.0 versus ≥1.0 mg/dL	2.187	0.266–17.98	0.467
ALBI grade, 1/2a versus 2b	2.875	0.667–12.40	0.157
ALBI score	9.083	1.118–73.76	**0.039**
BCLC stage, A/B versus C	1.785	0.332–9.608	0.500
Serum AFP, <100 versus ≥100 ng/mL	3.452	0.820–14.53	0.091
Serum DCP, <100 versus ≥100 mAU/mL	1.927	0.387–9.588	0.423
Esophageal varix, F0/1 versus F2/3*	N.A.	N.A.	N.A.
Treatment or rupture history of esophageal varix, yes versus no**	N.A.	N.A.	N.A.
DOAC administration, yes versus no	N.A.	N.A.	N.A.

*Note*: A bold number indicates the *p*‐value <0.05.

Abbreviations: AFP, α‐fetoprotein; ALBI, albumin‐bilirubin; ALT, alanine aminotransferase; APTT, activated partial thromboplastin time; AST, aspartate transaminase; CI, confidence interval; DCP, des‐γ‐carboxy prothrombin; DOAC, direct oral anticoagulant; HR, hazard ratio; N.A., not applicable; PT, prothrombin time.

* No patients with an F2/3 grade of esophageal varix had bleeding events.

** No patients with a treatment history of esophageal varix had bleeding events.

## 4 DISCUSSION

Patients are increasingly receiving Atezo/Bev combination immunotherapy‐based regimens, which pose a bleeding risk due to the inhibition of the VEGF pathway by bevacizumab, along with expanding their indications for unresectable HCC and advanced LC.^8^, ^9^ DOACs are also increasingly used because of their convenience.^3^, ^4^ Because both bevacizumab and DOACs have a bleeding risk, potential exacerbation of the bleeding risk due to Atezo/Bev‐based regimens in patients taking DOACs is a concern. For first‐line treatment of unresectable HCC, patients with contraindications for bevacizumab have been proposed for durvalumab and tremelimumab treatment^12^, ^13^; however, no detailed studies have been conducted to determine whether the administration of DOACs could be contraindicated for bevacizumab (i.e., bevacizumab‐unfit) due to the associated bleeding risks. Therefore, we investigated the feasibility of combining Atezo/Bev‐based regimens with DOACs for treating HCC and LC.

In the comparison of the baseline characteristics between the DOAC and NAA groups in this study, the PT was shorter in the DOAC group. This may be caused by the medicinal effect of DOAC that can lower the PT value.^16^ The occurrence rates of bleeding events were 9.1% and 10.8% in the DOAC and NAA groups, respectively, with no significant difference between the two groups. These rates were comparable to the 8.4%–23.0% rate in previous clinical trials of Atezo/Bev‐based regimens in unresectable HCC and advanced LC.^9^, ^17^ Besides, no significant difference was found in the frequency of bleeding events of grade ≥3 between the two groups. None of the patients in the DOAC group discontinued Atezo/Bev‐based regimens because of severe bleeding. Additionally, DOAC administration did not contribute to bleeding events using the multivariate analysis. These findings suggest that Atezo/Bev‐based regimens might be feasible in patients receiving DOACs.

Although the combination of Atezo/Bev‐based regimens and DOACs, both of which cause adverse bleeding events, may increase the risk of bleeding, this was not the case in the present study. A case series of ovarian cancer combining bevacizumab with DOAC showed similar findings.^18^ On the other hand, for tyrosine kinase inhibitors (TKIs) targeting the VEGF pathway, such as cabozantinib and sunitinib, studies have addressed the caution that their concurrent treatment with anticoagulant agents, including DOACs, in renal cell carcinoma harbored a greater risk of bleeding than did anti‐VEGF TKIs alone.^19^, ^20^ The bleeding risks concurrent with DOACs were different between recombinant monoclonal antibodies and TKIs, although both target the VEGF pathway. This may be due to the drug–drug interactions that depend on pharmacokinetics. DOACs have two main metabolic pathways: via the permeability glycoprotein (P‐gp) efflux transporter and cytochrome P450, including CYP3A4.^21^ TKIs can inhibit the cytochrome P450 and P‐gp pathways, which alter DOAC metabolism and increase its blood concentration. As a result, DOAC efficacy may be enhanced, and bleeding events may increase.^22^ In contrast, the metabolism of monoclonal antibodies depends on nonspecific catabolism in the reticuloendothelial system or its disappearance via binding to the target molecule (i.e., VEGF for bevacizumab).^23^, ^24^ Thus, the bleeding risk due to drug–drug interactions between bevacizumab and DOACs are unlikely to increase. However, since a recent report has highlighted that bevacizumab has a higher bleeding risk compared with TKIs in the patients receiving DOACs,^25^ further research on the bleeding risk in patients receiving combination of anti‐VEGF agents and DOACs is warranted.

Hypoalbuminemia was screened in all patients in the present study with respect to factors associated with bleeding events. This may be because exhaustion or malnutrition can lead to bleeding.^26^ In patients with HCC, a high ALBI score was associated with bleeding events. A low hepatic reserve may lead to a bleeding tendency due to decreased thrombopoietin production resulting in a low platelet count, or through a deficiency in coagulation factors.^27^

Our study has some important limitations, including its retrospective nature, single‐center design, and small sample size. Analysis was not possible for the therapeutic effects of Atezo/Bev‐based regimens because two different cancer types, HCC and LC, were analyzed simultaneously.

In conclusion, DOACs did not have a considerable effect on the bleeding risk in Atezo/Bev‐based regimens for HCC and LC. Therefore, Atezo/Bev‐based regimens may be feasible in patients taking DOACs under careful surveillance for bleeding, although further investigation is required to validate our results.

## AUTHOR CONTRIBUTIONS

**Tasuku Nakabori:** Conceptualization (lead); data curation (lead); formal analysis (lead); methodology (equal); resources (equal); writing – original draft (lead); writing – review and editing (lead). **Kei Kunimasa:** Conceptualization (equal); resources (equal); writing – original draft (equal). **Masaki Kawabata:** Resources (equal). **Sena Higashi:** Data curation (equal); formal analysis (equal); resources (equal). **Kaori Mukai:** Resources (equal). **Takahisa Kawamura:** Resources (equal). **Takako Inoue:** Resources (equal). **Motohiro Tamiya:** Resources (equal). **Kazumi Nishino:** Resources (equal). **Kazuyoshi Ohkawa:** Resources (equal); writing – review and editing (equal).

## FUNDING INFORMATION

The authors confirm that there are no funding sources to declare.

## CONFLICT OF INTEREST STATEMENT

The authors declare no conflicts of interest for this article.

## ETHICS STATEMENT

Approval of the research protocol: The protocol for this research project has been approved by a suitably constituted Ethics Committee of the institution, and it conforms to the provisions of the Declaration of Helsinki. Institutional Review Board for Clinical Research at Osaka International Cancer Institute, Approval no. 23200.

## INFORMED CONSENT

The Institutional Review Board for Clinical Research of Osaka International Cancer Institute (approval number: 23200) waived the requirement for informed consent. The opt‐out method was provided to the patients on our hospital's website.

## Supporting information


**Figure S1.** Cumulative incidence of bleeding events. DOAC, direct oral anticoagulant.

## Data Availability

The data shown in this study are available from the corresponding author upon reasonable request.
