# Supplementary material for: Feasibility of atezolizumab and bevacizumab combination regimens in patients with hepatocellular carcinoma and lung cancer taking direct oral anticoagulants
Source: Cancer Med. 2024 Jun 25;13(12):e7430. doi: 10.1002/cam4.7430 (PMC11196953; doi:10.1002/cam4.7430)
Supplement: Supplementary file 1 — Figure S1. Cumulative incidence of bleeding events. DOAC, direct oral anticoagulant. [file CAM4-13-e7430-s001.pptx]

## Slide 1
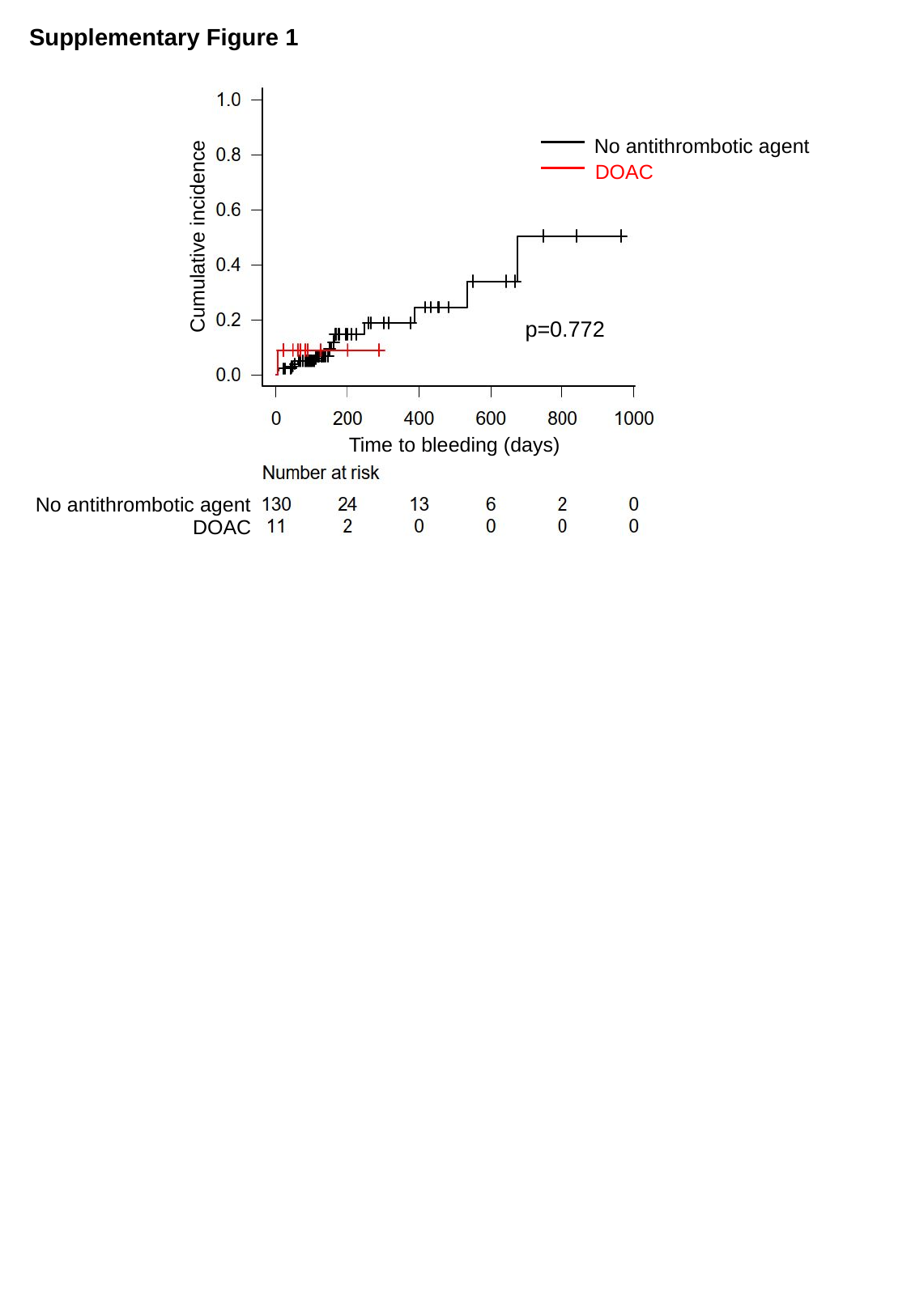

Supplementary Figure 1
No antithrombotic agent
DOAC
Cumulative incidence
p=0.772
Time to bleeding (days)
No antithrombotic agent
DOAC
